# Micro RNA-19a interferes with IL-10 expression in peripheral dendritic cells of patients with nasal polyposis

**DOI:** 10.18632/oncotarget.16555

**Published:** 2017-03-24

**Authors:** Xiang-Qian Luo, Jian-Bo Shao, Rui-Di Xie, Lu Zeng, Xiao-Xi Li, Shu-Qi Qiu, Xiao-Rui Geng, Li-Tao Yang, Lin-Jing Li, Da-Bo Liu, Zhi-Gang Liu, Ping-Chang Yang

**Affiliations:** ^1^ Department of Otolaryngology, Guangzhou Women and Children's Medical Center, Guangzhou Medical University, Guangzhou 510010, China; ^2^ The Research Center of Allergy and Immunology, Shenzhen University School of Medicine, Shenzhen 518060, China; ^3^ Longgang ENT Hospital, Shenzhen 518116, China; ^4^ Brain Body Institute, McMaster University, Hamilton, ON, L8N 4A6, Canada

**Keywords:** nose, nasal polyp, dendritic cells, micro RNA, interleukin-10

## Abstract

The pathogenesis of nasal polyp is to be further investigated. Micro RNA (miR) plays a role in the development of allergic inflammation. Interleukin (IL)-10-producing dendritic cells (DC) have immune tolerogenic properties. This study test a hypothesis that miR-17-92 cluster is associated with suppressing IL-10 in peripheral DC. In this study, peripheral blood samples were obtained from 26 patients with nasal polyp. The CD11c DCs were isolated from the blood samples and analyzed for the expression of IL-10. We observed that, as compared with healthy subjects, the IL-10 expression in peripheral DC was significantly lower in polyp patients. The levels of miR-19a, but not the rest 5 members of the miR-17-92 cluster, were markedly higher in DCs in polyp group. Exposure to recombinant IL-4 suppressed the IL-10 expression in DCs, which was abolished by blocking histone deacetylase-11 or knocking down the miR-19a gene in DCs. We conclude that miR-19a plays a critical role in the suppression of IL-10 in peripheral DCs, which may be a target in the immune therapy for nasal polyp.

## INTRODUCTION

Nasal polyps are polypoidal tissue arising from the nasal mucosal membranes and paranasal sinuses [1]. They frequently accompany allergic rhinitis [2]. Nasal polyps can be classified into two types, the antrochoanal polyps and the ethmoidal polyps. Antrochoanal polyps arise from the maxillary sinuses; ethmoidal polyps arise from the ethmoidal sinuses [1]. Clinical symptoms of nasal polyps include nasal congestion, chronic sinusitis, loss of smell, and headache [1]. The pathogenesis of nasal polyp is unclear, but most commonly thought to be caused by nasal allergy [2]. During allergic attacks, mast cells are activated to release chemical mediators, such as histamine, to induce mucosal edema and local inflammation, which play an important role in the development of nasal polyposis.

Nasal allergy is an IgE-mediated allergic inflammation of the nasal mucosa, featured by T helper (Th) 2 cell polarization [3]. As usual, the Th1 and Th2 immune response are tightly regulated by the immune regulatory system in the body and is maintained in a Th1/Th2 balance status [4]. Although Th1 or Th2 cells in the body contact foreign antigens constantly, the response to the foreign antigens by Th1 or Th2 cells is maintained in a given extent by immune regulatory cells. The immune regulatory cells mainly include regulatory T cells and regulatory B cells [5, 6]. Some sub-fractions of dendritic cells (DC) also have an immune regulatory function. IL-10 and transforming growth factor-β are the main immune regulatory mediators [7]. The deregulation of the immune regulatory system has been recognized in a large number of immune disorders, such as airway allergy [8], food allergy [9] and allergic dermatitis [10]. Yet, the mechanism of the immune tolerance breakdown is to be further investigated.

Micro RNAs (miR) are non-coding single stranded RNAs with 18-22 nucleotides in length [11]. Cumulative evidence indicates that miRs are associated with the pathogenesis of allergic disorders; including nasal allergy [12], asthma [13] as well as nasal polyp [14]. It is suggested that the miR-17-92 cluster can suppress the expression of IL-10 [15]. Since IL-10 is an important immune regulatory molecule, DCs are the critical cell population to initiate an immune reaction and to determine the immune response types, we hypothesize that miR-17-92 cluster may be involved in the deregulation of IL-10 expression in DCs. To test the hypothesis, we collected peripheral DCs from patients with nasal polyp and found that the expression of IL-10 in peripheral DCs was compromised, which was negatively correlated with the increase in miR-19a in DCs.

## RESULTS

### Patients with nasal polyp and allergic rhinitis show low levels of IL-10 in peripheral DCs

**Figure 1:**
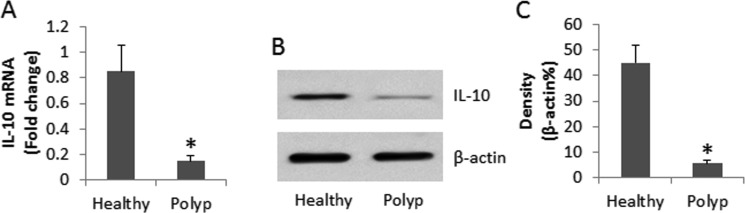
Assessment of IL-10 expression in peripheral DCs (**A**) the bars indicate the IL-10 mRNA levels, (**B**) the immune blots indicate the IL-10 protein levels in peripheral DCs. (**C**) the bars indicate the density analysis of B. DCs were isolated from blood samples collected from 26 polyp patients and 10 healthy subjects. Samples from individual subjects were analyzed separately. Data of bars are presented as mean ± SD. *p < 0.01, compared with the healthy group.

To understand the immune tolerant status of DC, we collected the peripheral blood samples from 26 patients with nasal polyp (polyp group). DCs were isolated from the blood samples and analyzed for the expression of IL-10. As compared with healthy controls, the IL-10 levels in DCs were significantly lower in polyp group (Figure 1). The data demonstrate that the allergic status of polyp patients affects the immune tolerant status of DC.

### High levels of miR-19a are detected in peripheral DCs in polyp patients

**Figure 2:**
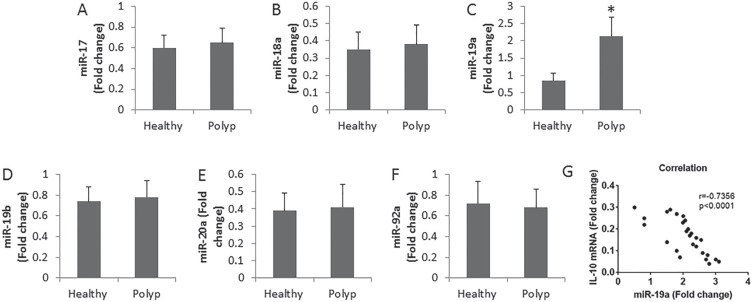
Assessment of miR-17-92 cluster in DCs (**A**–**F**) the bars indicate the levels of miR-17-92 cluster in DCs. The DCs were isolated from the peripheral blood samples of patients (*n* = 26) and healthy subjects (*n* = 10). Samples from individual subjects were analyzed separately. (**G**) the correlationship between IL-10 mRNA and miR-19a in DC of patients with nasal polyps. Data of bars are presented as mean ± SD. *p < 0.01, compared with the healthy group.

It was reported that miRs were involved in compromising the immune tolerant status [16]. We then assessed the levels of miR-17-92 cluster in the peripheral DCs. The results showed that the levels of miR-19a were markedly up regulated in DCs, while the levels of miR-17, miR-18a, miR-19b, miR-20a and miR-92a in DCs were not different between polyp group and healthy group (Figure 2A–2F). In addition, a negative correlation was identified between the expression of IL-10 and miR-19a in DCs in patients with nasal polyps (Figure 2G). The results imply that miR-19a may play an important role in the regulation of the immune tolerant status of DC.

### miR-19a mediates the effects of IL-4 on suppression of IL-10 expression in DCs

**Figure 3:**
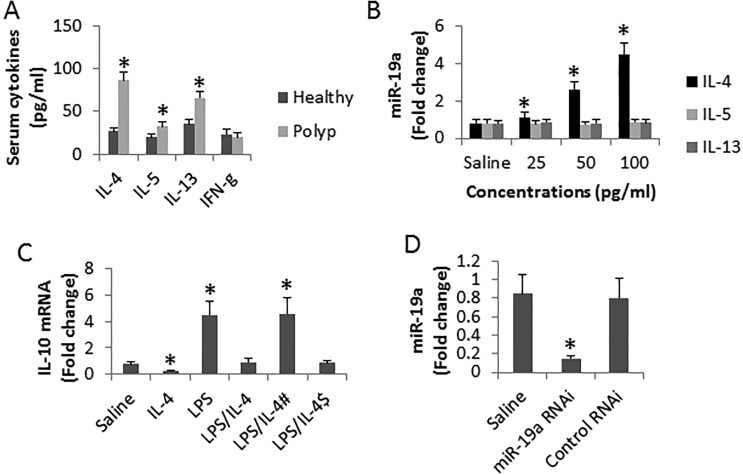
Assessment of the effects of IL-4 on suppression of IL-10 in DCs (**A**) the bars indicate the serum cytokine levels from 26 polyp patients and 10 healthy subjects. (**B**) the bars indicate the levels of miR-19a in DCs (cells were isolated from 3 healthy subjects) after exposing to Th2 cytokines in the culture for 48 h. (**C**) the bars indicate the levels of IL-10 mRNA in DCs (isolated from 6 healthy subjects) after the treatment as denoted on the X axis. IL-4: 100 pg/ml. LPS: 1 μg/ml. ^#^DCs were treated with miR-19a RNAi. ^$^DCs were treated with control shRNA. (**D**) the bars indicate the levels of miR-19a in DCs after treated with miR-19a RNAi or control shRNA. Data are presented as mean ± SD. *p < 0.01, compared with the healthy group (A) or saline group (B–D). The data are summarized from 3 independent experiments.

To find the possible causative factors of high expression of miR-19a in DCs, we assessed the cytokine levels in the serum. The results showed that the levels of Th2 cytokine, including IL-4, IL-5 and IL-4, were significantly higher in polyp group than in the healthy group, while the levels of IFN-γ we're not different from each other (Figure 3A). The results implicate that one of the Th2 cytokines might be the offending factor to up regulates the expression of miR-19a in DCs. To test this, we isolated DCs from blood samples of healthy subjects. The DCs were stimulated with recombinant Th2 cytokines at gradient concentrations in the culture for 48 h. The results showed that IL-4 significantly increased the expression of miR-19a in DCs, while no apparent effects of IL-4 and IL-5 on the expression of miR-19a were observed (Figure 3B). On the other hand, we tested the effects of IL-4 on the IL-10 expression in DCs. We treated DCs with LPS to up regulate the expression of IL-10, which was suppressed by the presence of IL-4 (Figure 3C). To elucidate if miR-19a mediates the effects of IL-4 on suppression of IL-10 in DCs, we knocked down the gene of miR-19a in DCs (Figure 3D). The miR-19a-deficient DCs were exposed to IL-4 and LPS. Indeed, the IL-4-induced IL-10 suppression was abolished (Figure 3C). The results indicate that IL-4 can suppress the IL-10 expression in DCs via up regulating miR-19a.

### HDAC11 mediates the effects of IL-4 on suppression of IL-10 in DCs

**Figure 4:**
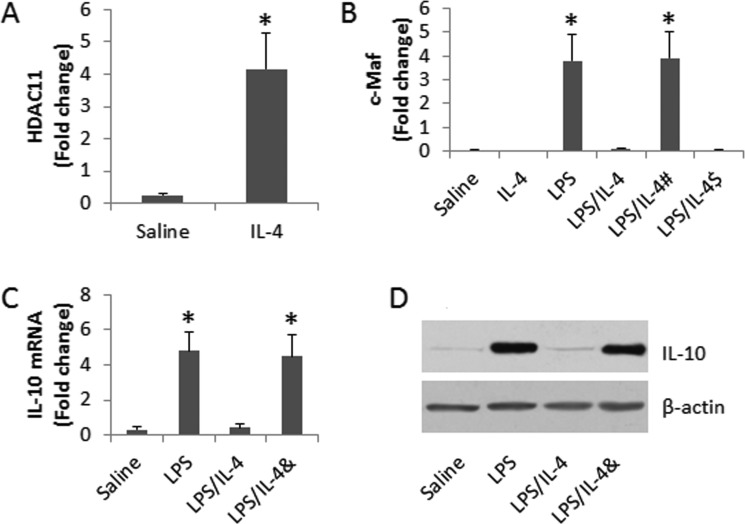
Assessment of the role of HDAC11 in the IL-4-suppressed IL-10 expression in DCs (**A**–**B**) the bars indicate the levels of HDAC11 (A) and c-Maf (B) at the IL-10 promoter locus in DCs. C-D, the results of IL-10 expression in DCs. The treatment of DCs is denoted on the X axis. LPS: 1 μg/ml. IL-4: 100 pg/ml. ^#^DCs were treated with miR-19a RNAi. ^$^DCs were treated with control shRNA. &, the presence of PCA (10 μM). Data are presented as ± SD. *p < 0.01, compared with the saline group. The data are representatives of 3 independent experiments.

Published data indicate that HDAC11 is involved in suppression of IL-10 [15]. To elucidate if HDAC11 was involved in the IL-4-suppressed IL-10 expression in DCs, we assessed the levels of HDAC11 at the promoter locus of IL-10 in DCs after exposing to IL-4 for 48 h in the culture. The results showed that the levels of HDAC11 at the IL-10 promoter locus were significantly increased (Figure 4A). To elucidate if exposure to IL-4 alter the activities of the IL-10 gene transcription factor, we assessed the status of c-Maf (an IL-10 gene transcription factor) at the IL-10 promoter locus in DCs after exposure to IL-4 in the culture, which was abolished by adding a HDAC11 inhibitor to the culture or knocking down the miR-19a gene in DCs (Figure 4B). The results indicate that HDAC11 mediates the effects of IL-4 on repressing the IL-10 gene transcription in DCs. To corroborate the results, we checked the IL-10 expression in DCs after exposure to IL-4 in the presence or absence of HDAC11 inhibitor (Figure 4C–4D).

## DISCUSSION

It is suggested that allergic response plays a critical role in the pathogenesis of a large part of nasal polyp. The mechanism is to be further investigated. The present data show a previously unknown phenomenon that peripheral DCs in patients with nasal polyp and nasal allergy express high levels of miR-19a and low levels of IL-10. Exposure to IL-4 up regulates the expression of miR-19a and suppresses IL-10 expression in DCs. The IL-4-suppressed IL-10 expression in DCs can be inhibited by the presence of HDAC inhibitor or knockdown of miR-19a. The data suggest that inhibition of miR-19a or HDAC inhibitor may have the therapeutic potential in the treatment of nasal polyp.

Besides the antigen presenting function, DCs also have the important immune regulatory capacity by producing IL-10 or TGF-β to induce regulatory T cells and regulatory B cells. Because of the immune regulatory feature, IL-10-producing DCs are designated as regulatory DCs [17]. Lobo et al found that regulatory DCs required IL-10 and programmed death 1 as well as down regulation of CD40 and p65 NF-κB phosphorylation to protect in renal ischemia/reperfusion injury. Our previous studies indicated that DCs produced TGF-β to be the tolerogenic DCs and had the capacity to induce regulatory T cells [18]. However, the factors interfering with IL-10 expression in DCs have not been well defined. The present data show that miR-19a may one of the factors to inhibit the expression of IL-10 in DCs of patients with nasal allergy and nasal polyp. Similar data were also reported by Simpson et al in which the authors found that higher levels of miR-19a facilitated the Th2 polarization [15].

A large body of evidence indicates that miRs are involved in the pathogenesis of immune inflammation. Nasal polyp is also an immune inflammation. Allergic response plays a role in the development of nasal polyp. In addition to having nasal polyp, the patients of this study also had nasal allergy. So far the pathogenesis of nasal polyp has not been well understood. The present data provide a possible hint that the increase in miR-19a and decrease in IL-10 in peripheral DCs in patients with nasal polyp may play a role in the development of nasal polyp. The precise mechanism needs further studies in animal models.

The miR-17-92 cluster is associated the pathogenesis of a large number of immune disorders, including cancer [19], allergy [15], diabetes [20], fibrosis [21], etc. In line with previous studies [15], we also found miR-19a was up regulated in patients with nasal polyp and allergic rhinitis. It is noteworthy that the increase in miR-19a negatively correlates with the decrease in IL-10 expression in peripheral DCs, implicating that miR-19a is a possible offending factor in the suppression of IL-10 in DCs. The inference is supported by subsequent data; knockdown of miR-19a inhibited the IL-4-induced IL-10 suppression in DCs.

HDAC11 is one of the members of the HDAC family. In general, HDAC acts as an inhibitory factor in gene transcription. The present study also showed that HDAC11 played a critical role in the IL-10 suppression induced by IL-4. The presence of HDAC11 inhibitor abolished the IL-4-suppressed IL-10 expression in DCs, indicating that the HDAC11 inhibitor may be a candidate in the regulation of immune tolerance by blocking the Th2 cytokine-compromised IL-10 expression in DCs.

In summary, the present study indicated that high levels of miR-19a and low levels of IL-10 were observed in peripheral DCs in patients with nasal polyp and allergic rhinitis. Blocking miR-19a or HDAC11 facilitated the expression in DCs.

## MATERIALS AND METHODS

### Patients

Nasal polyp patients were recruited into this study at the Department of Otolaryngology, Longgang ENT Hospital (Shenzhen, China). The diagnosis of nasal polyp and rhinosinusitis was carried out by our ENT surgeon based on the disease history, nasal cavity examination and CT scan. The diagnosis of nasal allergy was based on the disease history, nasal cavity examination, positive skin prick test and serum specific IgE levels were greater than 0.35 KU/L. Exclusion criteria: Asthma; nasal surgery history; complicated with severe rhinosinusitis; severe organ diseases; other autoimmune diseases and cancer. The demographic data are presented in Table 1.

**Table 1:** Demographic data

Parameter	Polyp group	Healthy group
**Male**	13 (50%)	5 (50%)
**Female**	13 (50%)	5 (50%)
**Age**	28.5 ± 7.6	28.8 ± 5.4
**Nasal allergy**	26 (100%)	0
**Surgery history**	0	0
**Maxillary sinusitis**	14 (53.8%)	0
**Front sinusitis**	0	0
**Ethmoid sinusitis**	0	0
**Sphnoid sinusitis**	0	0

Nasal allergy: 26 polyp patients were sensitized to house dust mite.

### Ethic statement

The using human tissue in the present study was approved by the Human Ethic Committee at Shenzhen University. The experimental procedures were carried out in accordance with the approved guidelines. An informed written consent was obtained from each subject.

### Collection of peripheral blood samples from human subjects and DC isolation

Twenty milliliter blood samples were collected from each human subject via ulnar vein puncture. The peripheral blood mononuclear cells (PBMC) were isolated from the blood samples with the gradient density centrifugation. The sera were collected for further experiments. CD11c^+^ MHC II^+^ DCs were purified from PBMCs with a DC isolation reagent kit (Miltenyi Biotech) following the manufacturer's instructions. The purity of DC was greater than 98% as checked by flow cytometry. The viability of the isolated DC was greater than 99% as assessed by Trypan exclusion assay.

### Cell culture

The isolated DCs were cultured in RPMI1640 medium supplemented with 10% fetal bovine serum, 100 U/ml penicillin, 0.1 mg/ml streptomycin and 2 mM glutamine. The cell viability was greater than 99% before using for further experiments as assessed by Trypan blue exclusion assay.

### Assessment of IL-10 mRNA and miR-17-92 cluster by real time RT-PCR

The total RNA was extracted from DCs with TRIzol reagents (Invitrogen). The cDNA was synthesized with the RNA using a reverse transcription kit (Invitrogen). RT-PCR was performed with the cDNA and SYBR Green Master Mix (Invitrogen) in a real time PCR device (Bio-Rad). The primers of miR-17-92 and IL-10 were provided by Enke Biotech (Shenzhen, China). The results were calculated with the 2^−ΔΔCt^ method and presented as fold change against RNA U6B (Invitrogen) (for miR-17-92 cluster) or control group (for IL-10). The primers of IL-10 include gccaagccttgtctgagatg and aagaaatcgatgacagcgcc.

### Assessment of IL-10 protein in DCs by Western blotting

The total proteins were extracted from DCs and quantified using the Bio-Rad protein assay. The protein was fractioned by SDS-PAGE and transferred onto a PVDF membrane. After blocking with 5% skim milk, the membrane was incubated with the anti-IL-10 antibody at 4°C overnight, washed with TBST (Tris-buffered saline Tween 20) for 3 times, incubated with a peroxidase-labeled second antibody for 1 h at room temperature, washed with TBST for 3 times. The membrane was developed with enhanced chemiluminescence. The results were photographed with an image station (UVI, Cambridge, UK). The density of the immune blots was determined by Photoshop software and presented as fold change against the internal control β-actin.

### Assessment of the serum cytokine levels by enzyme-linked immunosorbent assay (ELISA)

The serum was diluted to 50 folds and analyzed for the levels of IL-4, IL-5, IL-13 and IFN-γ with commercial reagent kits (R&D Systems) following the manufacturer's instructions.

### Knockdown of the miR-19a gene by RNA interference (RNAi)

The miR-19a gene was knocked down in DCs by RNAi. The RNAi reagent kit of miR-19a was provided by Beijing Yijie Biotech (Beijing, China). The miR-19a RNAi was performed with DCs following the manufacturer's instructions. The RNAi effect on DC was assessed by RT-qPCR.

### Preparation of nuclear extracts from DCs

The isolated DCs were incubated with lysis buffer at 4°C for 15 min, and centrifuged at 500 × g for 10 min at 4°C. The supernatant was collected using as cytosolic extract. The pellet was added with nuclear extract buffer and incubated for 15 min at 4°C, and centrifuged at 13,000 × g for 10 min at 4°C. The supernatant was collected as nuclear extract. The protein concentrations were determined by the Bradford method.

### Assessment of HDAC11 and c-Maf at the IL-10 promoter locus by chromatin immunoprecipitation assay (ChIP)

ChIP assay was performed with DCs using a reagent kit (Sigma Aldrich) following the manufacturer's instructions. Briefly, DCs were fixed with 1% formaldehyde for 15 min, lysed and sonicated to shear the chromatin DNA to 100–500 bp. Cell lysates were precleared by incubation with protein G-agarose beads for 2 h at 4°C. The supernatant was collected and incubated overnight at 4°C with 2 μg of antibodies of HDAC11 or c-Maf (Santa Cruz Biotech) or isotype IgG (a negative control). The antibody-chromatin complex was precipitated by incubation with protein G-agarose beads for 1 h at 4°C. The beads were centrifuged, washed and eluted in elution buffer. DNA was recovered from the precipitated samples by reverse crosslinking at 65°C for 4 h and digested with proteinase K for 1 h at 45°C to remove proteins, then the immunoprecipitated DNA was recovered by phenol/chloroform extraction and ethanol precipitation. The DNA or input was analyzed by PCR with the miR-19a promoter primers (Beijing Yijie Biotech; Beijing, China). The results were presented as folds of input.

### Statistical analysis

Data were presented as mean ± SD. Differences between two groups were determined by Student t test or using one-way ANOVA in more than two groups. Bonferroni test was used as a post hoc test after one-way ANOVA. The criterion of significance was set at *p* < 0.05.
